# Modeling the relationship between wolf control and cattle depredation

**DOI:** 10.1371/journal.pone.0187264

**Published:** 2017-10-30

**Authors:** Lyudmyla Kompaniyets, Marc A. Evans

**Affiliations:** 1 School of Economic Sciences, Washington State University, Pullman, Washington, United States of America; 2 Department of Mathematics and Statistics, Washington State University, Pullman, Washington, United States of America; Universita degli Studi di Sassari, ITALY

## Abstract

Wolf control to reduce cattle depredation is an important issue to ecology and agriculture in the United States. Two recent papers use the same dataset having wolf population characteristics and cattle depredation, but come to opposing conclusions concerning the link between wolf control and cattle depredation. Our paper aims to resolve this issue by using the same dataset and developing a model based on a causal association that would explain the nature of the relationship between wolf control and cattle depredation. We use the data on wolf population, number of cattle, number of wolves killed and number of cattle killed, from the U.S. Fish and Wildlife Services Interagency Annual Wolf Reports over the period of 1987–2012. We find a positive link between wolf control and cattle depredation. However, it would be incorrect to infer that wolf control has a positive effect on the number of cattle depredated. We maintain that this link comes from a growing wolf population, which increases cattle depredation, and in turn, causes an increase in the number of wolves killed. While the wolf population is growing, we see both wolf removal and cattle depredation simultaneously grow. It is not until the wolf population growth nears the steady state, that removal of wolves has a sufficient negative effect to reduce or stabilize the number of cattle depredated.

## Introduction

The issue of wolf control to reduce livestock depredation has an important place in agricultural and ecological literature. Removing wolves is generally thought to help ranchers reduce their livestock losses through a reduction in the wolf-cattle interactions. Two recent papers (Wielgus and Peebles [1], and Poudyal et al. [2]) use the same dataset to draw opposing conclusions about the effect of wolf control on cattle depredation. Our paper focuses on solving the same problem, and attempts to determine the true relationship between wolf control and cattle depredation.

Wolf control and its consequences are an important issue in the western United States. In reaction to depredations on livestock and wildlife, gray wolves (*Canis lupus*) were essentially extirpated from the western U.S. by the 1930s. In 1973, the gray wolf was given protection under the Endangered Species Act, which also laid the ground work for the required recovery of the species. In the early 1980s, Canadian gray wolves began colonizing the northwestern portion of Montana and by 1987, there were an estimated 10 gray wolves in Montana. In 1995, gray wolves were reintroduced to Yellowstone National Park located at the borders of Montana, Idaho and Wyoming. Seventeen years later, Montana, Wyoming and Idaho had an estimated 625, 277 and 683 wolves, respectively. The rapid recovery of wolves from near extinction in the 1980s to the current level of more than 1,500 wolves in the western US indicates the adaptability of these animals. See the U.S. Department of Justice web page for a brief history of the gray wolf in the western U.S. [3].

With the increase in wolf numbers in the western U.S., there has been a corresponding increase in cattle and sheep depredation due to wolves. In 1995, the year wolves were introduced into Yellowstone National Park, only three cattle and no sheep were killed by wolves in Montana, Wyoming and Idaho, while in 2012, Montana, Wyoming and Idaho lost 67, 44 and 73 cattle, respectively, while the same three states also lost 37, 112 and 312 sheep, respectively. From reintroduction until 2012, wolf removals due to depredation were mainly the duty of the US Fish and Wildlife Service or state wildlife agencies. In 2009, trapping and sport seasons for wolves were initiated in Montana, Wyoming and Idaho, though the gray wolf was subsequently relisted as an endangered species in Wyoming.

Wolf reintroduction to rural agricultural areas in North America and the resulting conflicts with the human population have received substantial attention in the wildlife and ecological literature [4, 5, 6, 7, 8]. These studies attempt to understand wolf population dynamics and their interplay with the habitat, as well as find ways to effectively manage wolf depredation. For example, a paper by Mech [9] gives an overview of wolf harvesting practices and provides suggestions on practices that align with wolf biology and public sensitivities towards wolf control.

Recent wildlife literature studies the effect of wolf control on wolf population recovery [8, 10, 11, 12]. Creel & Rotella argue that, against common belief, wolf removal is not compensatory, and an increase in wolf harvesting does not come with a decline in wolf natural mortality [10]. They find that while wolves can be sustainably harvested within limits, the effect of harvesting on wolf mortality can be super-additive, especially in the region of the Northern Rocky Mountains Recovery area. Murray et al. [11] study three wolf populations in northwestern United States (1982–2004) to evaluate the effect of anthropogenic mortality on natural demographic processes in wolves. The authors show that in expanding wolf populations, anthropogenic mortality is additive to wolf natural mortality. They also show the presence of compensatory processes when individuals are considered separately, based on their attitude to risk.

At the same time, literature on the methods of statistical modeling of predator-prey and predator-control data is rare. A paper by Musiani et al. [13] attempts to predict wolf depredation occurrence using monthly data from wolf depredation investigations for Alberta, Canada (1982–1996), for Idaho, Montana and Wyoming, USA (1987–2003). They find that wolf depredation and wolf control occur with a seasonal-annual pattern. Bradley et al. [14] use data from Montana, Idaho and Wyoming (1989–2008) to study and compare the effects of different wolf removal methods on livestock depredation occurrence and wolf recovery. The authors compare the effects of three management responses (no removal, partial pack removal, and full pack removal) on cattle depredation. The findings show that full removal is the most effective in reducing depredation, while partial removal has a different effect under different conditions.

Two recent papers produced by Wielgus and Peebles [1], and Poudyal et al. [2] highlight the difficulties in the modeling of predator-prey data and the interpretation of the estimated model parameters. Wielgus and Peebles [1], and a follow-up rebuttal paper by Poudyal et al. [2], attempt to develop statistical models to assess the long-term effectiveness of lethal wolf control on the prevention of livestock depredation. However, as we will explain, developing statistical models using standard statistical model selection procedures is fraught with pitfalls and great care should be exercised.

The data used in both papers are available from the US Fish and Wildlife Services Interagency Annual Wolf Reports [15] and cover the period of 1987–2012. These data were made available in the publication by Wielgus and Peebles [1] (these data are provided in a S1 Table of [1]). This data set is unique in that it tracks the colonization of wolves in the western U.S. from population initiation in the 1980s to a mature and relatively stable population in 2012. Outside of laboratory experiments, it is extremely rare to have data of this nature. Within this data set are seven (7) variables recorded for each year in each of Montana, Wyoming and Idaho: the number of cattle depredated by wolves, the number of sheep depredated by wolves, the minimum number of wolves in the population at the end of the year, the number of wolves killed (lethal control), the number of wolf breeding pairs, the number of cattle in the state, the number of sheep in the state, along with the year the data were recorded.

The aggregated nature of these data limits the possible research hypotheses that can be assessed, as cattle and sheep are not uniformly spread across each state, nor are the wolves. Conflicts between wolves and livestock can only occur where the ranges overlap. Of the approximately 2.5 million cattle in the state of Montana, about 18% are in the western portion of the state where wolves exist. Furthermore, not all wolf pack home ranges overlap lands with cattle. Thus, wolf removals typically occur in response to livestock depredations and only involve the wolves or wolf packs that committed the transgressions, while the majority of the wolf population has limited interaction with cattle. The models of Wielgus and Peebles [1], and Poudyal et al. [2] are based on state aggregated data, in which spatial loss of information has occurred. This, in turn, has likely led to less informative models.

Both Wielgus and Peebles [1], and Poudyal et al. [2] used a generalized linear model with a log-link function and negative binomial distribution to model cattle depredation counts as a function of the other variables. For count data of this nature, a statistical modeler would normally use a generalized linear model assuming a Poisson distribution [16, 17]. The use of a negative binomial distribution by both studies likely stems from a concern that the observed data are over-dispersed when compared to the Poisson distribution, yet this assumption did not appear to be assessed in either paper. In addition to the negative binomial distribution, one could also account for the over-dispersion by using a generalized linear mixed model, where any number of continuous mixing distributions might be used. In fact, the negative binomial distribution is the marginal distribution formed from the mixture of the Poisson distribution with a gamma mixing distribution. Statistical packages (e.g., SAS and R) are mostly limited to a normal mixing distribution. The use of other mixing distributions requires a substantial knowledge of statistics and programming, and as such, is outside the capacity of most researchers. In any case, Wielgus and Peebles [1], and Poudyal et al. [2] should likely have assessed the need for using the negative binomial distribution for modeling the data.

Wielgus and Peebles [1] used forward variable selection to develop a model to determine the relationship between livestock depredation (dependent variable) and a set of lagged independent variables, along with interaction terms. The lagged variables represent the value of each variable from the previous year and are commonly used in models with serially correlated data. Among the models developed by Wielgus and Peebles [1], the lowest AIC (464.02) is associated with the following model structure:

$$\begin{array}{l} Log(Cattle\_depredated_{t})=\beta_{0}+\beta_{1}Cattle_{t-1}+\beta_{2}Wolf\_Breeding\_Pairs_{t-1}\\ +\beta_{3}Wolves\_Killed_{t-1}+\beta_{4}(Wolf\_Breeding\_Pairs_{t-1}\times Wolves\_Killed_{t-1}) \end{array}$$

where t indexes time (year) and t-1 represents a variable lagged by one year.

For the Wielgus and Peebles [1] model, all model parameters are significant (P < 0.001), and all parameter estimates are positive, except for the interaction term. In addition, Wielgus and Peebles [1] include the variance inflation factors (VIF) for each predictor, and found moderate multicollinearity among the predictor variables, indicating that multicollinearity should not present a problem to the analysis. The actual VIF (subsequently calculated by the authors) are 1.44, 4.02, 5.22 and 2.65 for the predictors as they appear in the model. These values indicate moderate multicollinearity among some of the predictor variables. Multicollinearity can potentially reduce the statistical power associated with tests of the model parameters, but more importantly it can have a negative effect on the interpretation of the parameter estimates (e.g., sign changes of the parameter estimates between competing models).

By way of the significant positive parameter estimate for the number of wolves killed, Wielgus and Peebles [1] determine that removal of wolves shows a positive relationship with the number of cattle depredated. This conclusion is contrary to the general consensus of the wildlife research community that removal of wolves will have a negative effect (lowering) on the number of cattle depredated [9, 10, 18].

Poudyal et al. [2] replicated Wielgus and Peebles' [1] study by reanalyzing the data with the same generalized linear model approach, but with a different set of predictor variables. Poudyal et al. [2] correctly realize that when dealing with time sequence data, a variable for time should at least be considered as a part of the model, and that other time dependent variables (e.g., the number of cattle depredated, etc.) may require lagged versions of the variables since the values of these variables from the previous year may affect the response in the current year. In addition, Poudyal et al. [2] also included misspecification tests to assess the model assumptions of independent observations, log-linear model form and temporal homogeneity, all of which were shown to be satisfied by their model.

The Poudyal et al. [2] model has an AIC of 417.94 and has the following form:

$$\begin{array}{l} Log(Cattle\_depredated_{t})=\beta_{0}+\beta_{1}Year_{t}+\beta_{2}Cattle\_Depredated_{t-1}+\beta_{3}Wolf\_Breeding\_Pairs_{t}\\ +\beta_{4}Wolf\_Breeding\_Pairs_{t-1}+\beta_{5}Wolves\_Killed_{t}+\beta_{6}Wolves\_Killed_{t-1}+\beta_{7}Cattle_{t}+\beta_{8}Cattle_{t-1} \end{array}$$

where t indexes time (year) and t-1 represents a variable lagged by one year.

Only the time index, the lagged number of cattle depredated and the lagged number of wolves killed are significant (P < 0.001). While the first two predictors were positively significant, the lagged number of wolves killed was negatively significant. All other predictors were not significant at the 0.05 level. Based on these results, Poudyal et al. [2] conclude that the number of wolves killed is negatively related to the number of cattle depredated, a conclusion that is opposite to that of Wielgus and Peebles [1]. In this paper, we attempt to elucidate the relationship between wolf control and cattle depredation.

## Materials and methods

In this paper, we reanalyze the data presented in Wielgus and Peebles [1] (these data are provided in a S1 Table of the original paper [1]). We find that the papers of Wielgus and Peebles [1], and Poudyal et al. [2] have substantial issues with the statistical models developed. First, neither Wielgus and Peebles [1], nor Poudyal et al. [2] control for differences in the number of cattle depredations between the three states. Simple graphing of the number of cattle depredations against the year of recording will produce distinct differences between the three states. Second, the use of lagged variables by both Wielgus and Peebles [1], and Poudyal et al. [2] may be considered, but are not justified in this instance. In a given year, cattle depredations are a direct function of the number of wolves in the population, and the number of wolves killed is a direct function of the number of cattle depredated. Lastly, neither of the papers account for the nonlinear (sigmoidal) pattern in wolf population growth, cattle depredations and the number of wolves killed over time. Poudyal et al. [2] do include graphs of the cattle depredation, number of wolves, number of wolves killed and the number of breeding pairs plotted against the year of recording. These graphs clearly show a sigmoidal nonlinear growth, which is consistent with a series of linked predator-prey models: cattle depredations increase with increasing wolf population (predator of cattle), which in turn increases the predation of wolves (prey) by humans (predator of wolves). Thus, any model considered should include components that account for the nonlinear nature of the data. The log-link function used by Wielgus and Peebles [1], and Poudyal et al. [2] can only fit data with a sigmoidal form over a very narrow range and will have difficulty fitting the beginning and ending phases of the data (e.g., onset of wolf colonization and the phase where the wolf population has reached the steady state. The sigmoidal shape can be approximated when using a log-link function by using polynomial functions of time (e.g., Year, Year^2^, Year^3^, etc.).

So as not to deviate from the problem at hand, the model developed in this paper stays within the bounds of a generalized linear model with a log-link function and a negative binomial distributional assumption. Further discussion of the analysis using linked interdependent nonlinear predator-prey models will be left for another paper.

In our paper, the number of cattle depredated (in log-link form) is represented as a linear function of the state (Montana, Idaho, Wyoming), year, year^2^, year^3^, state by year interactions, state by year^2^ interactions, state by year^3^ interactions, number of wolves killed, and the interaction between the number of wolves killed and year. The proposed model is based on the approximate sigmoidal form of the three variables of interest: the number of cattle depredated, and the number of wolves killed with the year of observation, along with the interaction between the wolves killed and year. All computations presented in this paper were performed using the SAS statistical software [19]

The model form is as follows:

$$\begin{array}{l} Log(Cattle\_depredated_{st})=\beta_{0}+\beta_{1}Wolves\_killed_{t}+\beta_{2}(Wolves\_killed_{t}\times Year_{t})\\ +\beta_{3}Year_{t}+\beta_{4}Year_{t}^{2}+\beta_{5}Year_{t}^{3}+\beta_{6,s}State_{s}+\beta_{7,s}(State_{s}\times Year_{t})\\ +\beta_{8,s}(State_{s}\times Year_{t}^{2})+\beta_{9,s}(State_{s}\times Year_{t}^{3}) \end{array}$$

where State is an indicator variable for each state (Montana, Idaho, Wyoming) and is indexed by s, and t indexes the time period (year).

The above model does not include the wolf population variable and it is reasonable to ask why? Cattle depredation in a given year is caused by wolves, and is therefore, a function of the existing wolf population in the same year. As stated earlier, cattle are located in most areas of Montana, Wyoming and Idaho, but wolves exist over a much smaller portion of each state; there are areas in each state where cattle exist, but there are no wolves, and there are areas in each state where wolves exist, but there are no cattle. Thus the overall wolf population for a state is not a necessarily the best predictor of the number of cattle depredations.

More importantly, because cattle depredations lead directly to removal of the wolves involved [15], the number of wolves killed is likely a better predictor of cattle depredations. Furthermore, the results of both Wielgus and Peebles [1], and Poudyal et al. [2] were focused on the relationship between the number of cattle depredated and the number of wolves killed, so including wolves killed in the model makes sense. However, including both variables (wolf population size and wolves killed) in the model would lead to severe multicollinearity problems, which may adversely affect the significance of the parameter estimates. For all of these reasons, we chose not to include the variable wolf population in our model.

In order to assess the model assumptions, we develop a series of model diagnostics. These include McFadden’s R-squared, Efron’s R-squared, PRESS statistics, VIF, Durbin-Watson statistics and the Pearson residuals plotted against time.

Poudyal et al. [2] use McFadden’s R-squared to compare the fit of their model and the model proposed by Wielgus and Peebles [1]. McFadden’s R-squared, while a useful measure of model fit, is not easily interpreted. R-squared in linear regression models is bounded between 0 and 1, with 1 indicating a perfect fit between the model predictions and the observed data. McFadden’s R-squared, while having a lower bound of 0, does not typically approach a value of 1 and therefore some loss of interpretation is associated with it. Efron’s R-squared is simply the squared correlation between the observed response data and the model predicted values; hence it is bounded between 0 and 1, with a value of 1 implying perfect model fit. This makes Efron’s R-squared exactly equivalent to the R-squared of linear regression models.

Of course, R-squared statistics do not necessarily indicate whether a model is a good predictor of future observations, only that the model may be a good predictor of the data used to develop the model. PRESS statistics provide a much better indication of a proposed model to predict future observations. PRESS is equivalent to the computed sum of squared errors (e.g., sum of the squared difference between the observed response and the model predicted response), but with the predicted response for the i^th^ observation computed with the model parameters estimated when the i^th^ observation is removed from the data. PRESS provides information on the quality of model fit for future observations, which R-squared statistics do not necessarily provide [20].

VIF statistics were computed for each predictor variable in order to assess the potential impact of multicollinearity among the predictors. Multicollinearity can have two potentially negative impacts. First, it can adversely affect the variance associated with estimated model parameters and thereby lower the power of associated tests. Secondly, multicollinearity can, but may not necessarily, negatively impact the interpretation of a parameter estimate by changing the sign and size of the parameter estimate. Researchers should determine the sign of the parameters by first assessing the relationship between the response and each predictor individually. In the presence of strong multicollinearity, parameter estimates may vary in significance between similar models with predictors that are common to different models, and may also result in the loss of meaningful interpretation of the parameter estimates. Of greater concern would be a sign change in the parameter estimate between similar models. The authors believe that the strong multicollinearity among model predictors led to the different conclusions of Poudyal et al. [2] compared to those of Wielgus and Peebles [1]. In addition to multicollinearity among the model predictors, the number of cattle depredated is observed over time, thus, there is the potential for serial correlation in the model errors. Serially correlated errors would be a violation of the assumption of independent observations and can also result in lower power of the tests associated with the model parameters. The Durbin-Watson statistic can be used to assess whether the errors are serially correlated. Lastly, it is always a good idea to plot either the deviance or Pearson residuals against the model predictors (time) to assess the effects of extreme observations.

## Results

We begin by estimating the correlations and partial correlations between cattle depredation and the primary predictor variables (Table 1). The correlations clearly show the strength of the relationships. For Montana and Idaho separately, the correlation between cattle depredation and wolf population, wolves killed or breeding pairs generally exceed 0.90, while for Wyoming the correlations are moderately high. If you control for the effect of states, year, year^2^ and year^3^, the correlations remain moderately high, except that number of cattle in the states becomes nonsignificant. Although not shown, none of the one year lagged predictors had a higher correlation than the current year predictor, indicating that the non-lagged versions of the primary predictors were more highly related to the number of cattle depredated when compared to the lagged version.

**Table 1:** Estimated correlations and partial correlations between cattle depredation and the primary predictor variables.

	Predictor				
Method	Wolf Population Total	Wolf Population at Year End	Number of Wolves Killed	Number of Breeding Pairs	Number of Cattle
Montana Only	0.959 (<0.0001)	0.943 (<0.0001)	0.964 (<0.0001)	0.962 (<0.0001)	-0.621 (0.0009)
Wyoming Only	0.689 (0.0016)	0.671 (0.0023)	0.718 (0.0008)	0.669 (0.0024)	-0.723 (0.0010)
Idaho Only	0.927 (<0.0001)	0.915 (<0.0001)	0.980 (<0.0001)	0.810 (<0.0001)	-0.868 (<0.0001)
Controlling for States	0.848 (<0.0001)	0.8288 (<0.0001)	0.878 (<0.0001)	0.818 (<0.0001)	-0.679 (<0.0001)
Controlling for States, Year, Year^2^ and Year^3^	0.533 (<0.0001)	0.461 (0.0008)	0.644 (<0.0001)	0.389 (0.0053)	-0.113 (0.3335)

Our proposed model demonstrates a much improved fit over the models of Wielgus and Peebles [1] and Poudyal et al. [2], in part by having a lower AIC of 412.66. More importantly, when assessing the state variable as a single two degree of freedom test, along with each of its associated interactions with year, all terms of this model are highly significant (P < 0.05). This was not the case for the model proposed by Poudyal et al. [2]. The likelihood ratio statistics and associated p-values for the proposed model are shown in Table 2. Models with even lower AIC values are easily constructed by adding predictors to the proposed model. However, adding predictors to lower AIC would require the abandonment of model building based on logical ecological principles. Additionally, this would not necessarily improve upon the predictive nature of the proposed model, and would result in non-significant terms in the model that are hard to interpret due to multicollinearity.

**Table 2:** Likelihood ratio chi-square statistics and p-values for the proposed model of Kompaniyets and Evans.

Model Component	df	LR Chi-square	Pvalue
State	2	31.20	< 0.0001
Year	1	28.95	< 0.0001
Year^2^	1	24.18	< 0.0001
Year^3^	1	6.05	0.0139
Year*State	2	25.57	< 0.0001
Year^2^*State	2	20.25	< 0.0001
Year^3^*State	2	15.52	0.0004
Wolves Killed	1	21.54	< 0.0001
Year*Wolves Killed	1	18.49	< 0.0001

The parameter estimates for the proposed model appear in Table 3. For this model, all parameter estimates have appropriate signs (+ or -), as indicated by the estimated correlations shown in Table 1.

**Table 3:** Parameter estimates and estimated standard errors for the proposed model.

Predictor	Parameter Estimate	Est. SE
Intercept	-10.313	5.276
State		
- MT	10.437	5.266
- WY	-33.469	11.161
- ID	0.000	0.000
Year	2.007	0.923
Year^2^	-0.113	0.052
Year^3^	0.002	0.001
Year by State		
- MT	-1.652	0.916
- WY	4.984	1.804
- ID	0.000	0.000
Year^2^ by State		
- MT	0.084	0.051
- WY	-0.232	0.095
- ID	0.000	0.000
Year^3^ by State		
- MT	-0.001	0.001
- WY	0.003	0.002
- ID	0.000	0.000
Wolves Killed	0.119	0.024
Year by Wolves Killed	-0.005	0.001

The results presented in Tables 2 and 3 show a positive significant link between cattle depredation and the number of wolves killed. The parameter estimate for wolves killed is significant and positive (0.119), indicating that as more wolves are removed, the number of cattle depredated increases, much as Wielgus and Peebles [1] indicated. However, the interaction between the number of wolves killed and the year is significant and negative (-0.005), so that as time passes, the positive effect of wolves killed on cattle depredation decreases at the rate of -0.005 per year. Thus, by the 24^th^ year the effect of wolves killed on cattle depredation changes from positive to negative. However, this does not tell the entire story. In fact, the truth is somewhat difficult to tease out of this model, or the other models, because of the nonlinear nature of the data that is only being approximated by the proposed model.

The population of colonizing wolves grew modestly at first (lag phase). During this time there was little interaction between the relatively small population of wolves and cattle. However, a few years into colonization, the wolf population entered the exponential phase of population growth. During this phase, cattle depredation by wolves increased with the strongly increasing wolf population and the removal of the wolves that committed the transgressions subsequently increased. However, the rate of wolf removal was more than offset by the rate of wolf population growth. Although wolves are being removed at ever-increasing numbers, the number of cattle depredated is still increasing (positive relationship between cattle depredation and wolves killed). It is not until the wolf population nears the steady state of population growth at about year 24, that removal of wolves has a sufficient negative effect (negative interaction between wolves killed and year) to reduce the number of cattle depredated relative to prior years. Thus, the appearance that removing wolves has a positive effect on the number of cattle depredated is not true.

Table 4 presents the correlation coefficients (McFadden’s and Efron’s), PRESS statistic and the Durbin-Watson statistics for all three models (model of Poudyal et al. [2], Wielgus and Peebles [1] and the model proposed in this paper). In addition, these same statistics were computed for the data set with the observations from Wyoming in 2006 and 2007 removed, as these observations were determined to be highly influential on model fit. By all measures (AIC, R-squared, SSE, PRESS and Durbin-Watson), our proposed model outperforms the models of Poudyal et al. [2] and Wielgus and Peebles [1].

**Table 4:** Diagnostic statistics for the proposed model, the model of Wielgus and Peebles [1] and the model of Poudyal et al. [2].

Method	Poudyal et al.	Wielgus and Peebles	Kompaniyets and Evans
McFadden’s R^2^	0.266	0.167	0.294
Efron’s R^2^	0.534	0.660	0.890
Press Statistic	475,257.55	26,135.18	11,762.87
Press Statistic w/ Wyo. 2006 and 2007 removed.	11,259.84	18,153.14	6,654.32
Pvalues for the Durbin-Watson statistic to order 4			
Order 1	0.7821	0.9998	0.4901
Order 2	0.9621	0.9992	0.7483
Order 3	0.3790	0.8177	0.1209
Order 4	0.0296	0.0855	0.4011

The value of Efron’s R^2^ (Table 4) for the proposed model is much higher (0.89) than the models proposed by either Wielgus and Peebles [1] (0.66) or Poudyal et al. [2] (0.53), as are the values for McFadden’s R^2^. The correlations and model R^2^, in conjunction with the Press statistics, indicate that the proposed model is a much better predictor of cattle depredations than either of the other two models. It is worth noting that simple plotting of the deviance residuals over time resulted in detection of at least one very strong outlier. This outlier occurred in 2006 for the Wyoming data, where cattle depredation took a value of 124. All values of cattle depredation prior to and following this year did not exceed 54. To determine the effect of this outlier, the Press statistic was recomputed, but with the observation for 2006 (2007 lagged year) and 2007 removed. The Press statistic for the model of Poudyal et al. [2] dropped from 475,258 to 11,260, while the model of Wielgus and Peebles [1], went from 26,135 to 18,153, and the model proposed in this paper had a change in Press statistic of 11,773 to 6,654. The influence of this one observation was substantial for the model of Poudyal et al. [2] but less so for the models of Wielgus and Peebles [1] or the proposed model.

Poudyal et al. [2] used misspecification tests in an attempt to assess violations of the model assumptions. The components assessed included homogeneity of the time sequence and non-linearity of the functional form. The pvalues for the first through fourth order Durbin-Watson statistics are shown in Table 4. These values indicate that the model proposed by Poudyal et al. [2] has some residual serial correlation, as indicated by the significant fourth order statistics (P = 0.0296). The anticipated nonlinear shape of the relationship between cattle depredation and time was not detected by the misspecification tests. For the model proposed in this article, the nonlinear structure was approximated using a third order polynomial function and all components were found to be highly significant (P < 0.02), as shown in Table 2. Furthermore, not controlling for differences in the response due to the different states was also missed by the misspecification tests (Table 2). Thus, the misspecification tests provided by Poudyal et al. [2] had very low power for detection of the elements being assessed and may have resulted in the authors selecting a questionable model.

The VIF values are presented in Table 5. These indicate that there is substantial multicollinearity among the predictor variables for models by Poudyal et al. and Kompaniyets and Evans. Thus, some care should be taken when interpreting the estimated model parameters.

**Table 5:** Variance inflation factors (VIF) for mean centered model predictors of Wielgus and Peebles [1], Poudyal et al. [2], and Kompaniyets and Evans.

	Variance Inflation Factors		
Predictor	Wielgus and Peebles	Poudyal et al.	Kompaniyets and Evans
Cattle Depredated(t-1)	1.44	5.20	
Breeding Pairs(t)		9.54	
Breeding Pairs(t-1)	4.02	10.21	
Wolves Killed(t)		6.94	36.63
Wolves Killed(t-1)	5.72	7.68	
Wolves Killed(t-1)*Breeding Pairs(t-1)	2.65		
Number of Cattle(t)		119.05	
Number of Cattle(t-1)		120.48	
State- MT			3.31
- WY			2.92
- ID			
Year		4.21	61.35
Year^2^			6.37
Year^3^			45.87
Year by State- MT			11.63
- WY			7.69
- ID			
Year^2^ by State - MT			4.80
- WY			2.81
- ID			
Year^3^ by State - MT			29.50
- WY			7.29
- ID			
Year by Wolves Killed(t)			23.53

The model presented in this paper was chosen based on ecological principles and is consistent with the fundamental ecological literature. It also outperforms the models by Wielgus and Peebles [1] and Poudyal et al. [2] based on various measures of fit. Our results show a positive relation between wolf control and cattle depredation, as well as a negative interaction effect between year and wolves killed. While the wolf population is in the growth phase, cattle depredation will continue rising, despite the increasing numbers of wolves killed. However, when the wolf population reaches a more stable phase, wolf control efforts cause a reduction in the wolf population and, subsequently, the number of cattle depredated.

## Discussion

Modeling is as much art as it is statistical science. For designed experiments, where all variables are highly controlled, the correct statistical models are dictated by the design structure. Aside from meeting the model assumptions, the analyses for designed experiments are usually straightforward. On the other hand, developing models where there is no known a priori structure presents the researcher with substantial difficulties. If the goal is to develop models that predict the response well, then standard statistical model building methods, such as forward selection, stepwise selection, or best AIC based models will typically perform well. This is true of the models developed by Wielgus and Peebles [1], Poudyal et al. [2], and the proposed model. However, if one desires not only to predict the response, but also interpret the estimated model parameters, then much care must be taken so as not to put too much weight on these interpretations. It is always best to develop models based on a clear understanding of which predictors are causally related to the response and how these predictors should interact with the response.

The data set presented in Wielgus and Peebles [1], and used to develop the model proposed in this article, presents a rare opportunity. First, it is highly unusual to obtain predator data for a natural population that is observed from the onset of colonization through the steady state phase. Second, it is also unusual to have a data set where the relationships between the response, in this case the number of cattle depredated by wolves, has a clear set of causally related predictor variables, which include the minimum number of wolves in the population, the number of wolves killed, and how all of these variables change over time. Each of these variables is a sigmoidal-shaped nonlinear function of time. Furthermore, these variables are interrelated, so that changes in one variable will directly affect the others.

The model presented by Wielgus and Peebles [1] used forward selection to develop the model structure. As indicated earlier, model building techniques such as forward selection will typically produce models with reasonable predictive capabilities, but not necessarily model structures which are interpretable. Poudyal et al. [2] did not appear to use a standard model building technique, nor was their model developed based in ecological principles. Because of this and other problems already outlined for both Wielgus and Peebles [1] and Poudyal et al. [2], interpretation of the model parameters is questionable. In fact, if states and the components for a third order polynomial (to approximate the nonlinear structure of the data) are added to the model of Poudyal, et al. [2] the parameter estimate for the lagged predictor for wolves killed changes from negative, as shown in their manuscript, to positive. Why is this important? Because the sign of this parameter estimate was implied by Poudyal et al. [2] to indicate that the killing of wolves did indeed reduce cattle depredations. Wielgus and Peebles [1] did not include the number of wolves killed in their model, but did include the lagged predictor for wolves killed. They found the parameter estimate for this predictor to be positive and thus concluded that the removal of wolves actually increased the number of cattle depredated.

Our proposed model was deduced from fundamental ecological principles, although the model only approximates the nonlinear nature of the data, as previously indicated. It has a simple structure that produces the highest predictive value among the three models considered. Because of these two attributes, and because the sign on the parameter estimates for the model remains the same as the sign on the parameter estimates for the models having only a single predictor, interpretation of the estimated model parameters is warranted. Our results show a positive significant link between cattle depredation and the number of wolves killed. This finding is consistent with that of Wielgus and Peebles [1], although our interpretation of this result differs.

The effect of wolf removals on reducing cattle depredations only becomes apparent when the wolf population growth closes in on the steady state. This poses a dilemma for wolf managers. Removing wolves that depredate cattle will slow the relative rate of cattle depredations. However, cattle depredations will increase until the wolf population approaches a stable level. Only an increased removal of wolves well above and beyond the rate used by wildlife managers will reduce the rate of cattle depredations, but this level of removal is likely to increase public reaction to the killing of wolves. In fact, Wielgus and Peebles [1] did indicate that “Depredations increased with increasing wolf mortality up to about 25% mortality but then depredations declined when mortality exceeded 25%.” This statement is essentially correct. However, a more correct statement would indicate that the depredations will grow with increasing wolf mortality, so long as the wolf population is also growing at a rate exceeding the wolf population losses due to removals and natural mortality. If wolf population growth remains positive and the positive surplus is not offset by a corresponding mortality of wolves, cattle depredations will, on average, increase.
